# Palliative Homecare in Chronic Liver Disease: A Cohort Analysis of Factors and Outcomes Associated with Home Palliative Care in Patients with End-Stage Liver Disease

**DOI:** 10.1177/08258597241296116

**Published:** 2024-11-14

**Authors:** Hugo M. Oliveira, Céu Rocha, Maria Francisca Rego, Rui Nunes

**Affiliations:** 1 Department of Social Sciences and Health, Faculty of Medicine, University of Porto, Porto, Portugal; 2 Palliative Care Unit, Matosinhos Local Health Unit, Matosinhos, Portugal

**Keywords:** palliative care, hospice care, hepatology, end-stage liver disease, liver cirrhosis, terminal care

## Abstract

**Objective:** The prevalence and mortality of chronic liver disease has risen significantly. In end-stage liver disease (ESLD), the survival of patients is approximately 2 years. Despite the poor prognosis and high symptom burden, integration of palliative care in ESLD is reduced, and the majority of patients continue to die in inpatient care. We aim to assess predictors and outcomes of home palliative care, as well as factors associated with death at home in patients with ESLD. **Methods:** Retrospective cohort study of patients with ESLD, followed by a palliative care team between 2017 and 2022. Information regarding patient demographics, ESLD etiology, decompensations, and interventions was collected. Two-sided tests were used to identify factors associated with home palliative care. **Results:** We analyzed 75 patients: 44% had home palliative care and 33% died at home. ESLD patients with home palliative care were older (72.52 vs 64.45; *p* = 0.002), had a longer palliative care intervention time (149.97 ± 196.23 vs 43.69 ± 100.60 days; *p* = 0.007), higher rates of ascites or hepatic encephalopathy (χ^2^ = 11.024; *p* = 0.029), and hepatocarcinoma (90.9% vs 64.3%; *p* = 0.007). Patients with home palliative care had a reduction in-hospital admissions (2.61 vs 1.06; *p* = 0.000) and a greater probability of death at home (66.7% vs 33.3%; *p* = 0.000). Patients who died at home (33.3%) were older (72.20 vs 64.40; *p* = 0.000) and had longer palliative care intervention time (178.80 ± 211.78 vs 46.28 ± 99.67 days; *p* = 0.006). **Conclusion:** Home palliative care in ESLD differs based on demographics and disease complications, with a positive impact of homecare translated into a reduction in hospital admissions and an increased probability of death at home.

## Introduction

The prevalence and mortality of chronic liver disease have risen significantly worldwide in the last decades.^1,2^ Portugal shows this same trend, with death from liver disease ranking eighth and being the European country with the highest mortality from hepatocarcinoma.^3^ Chronic liver disease typically progresses from a compensated stage to the emergence of one or more complications associated with cirrhosis.^4^ These complications may include ascites, spontaneous bacterial peritonitis, hepatic encephalopathy, gastrointestinal bleeding, and hepatorenal syndrome, a phase designated as end-stage liver disease (ESLD).^4^ The survival of patients with ESLD is approximately 2 years, and the appearance of hepatocarcinoma can accelerate the course of the disease and worsen the prognosis.^4^ Patients with ESLD often experience a considerable symptom burden, including pain, fatigue, sleep disturbances, nausea, depression, and anxiety.^5^ They also have high rates of hospital readmissions^6,7^; a higher proportion are readmitted to the hospital within 30 days.^6^ As well as substantial healthcare costs and elevated rates of in-hospital mortality.^8,9^

Palliative care is a specialized medical care that addresses the physical, spiritual, and psychosocial needs of patients with serious illness and their caregivers.^10^ Emerging evidence suggests that palliative care can improve symptoms and enhance the quality of life for patients with ESLD.^11-13^ There is also evidence that early integration of palliative care reduces resource utilization among patients with cirrhosis^14,15^ and decreases the frequency of in-hospital deaths.^16^ Despite the growing research^17,18^ and the recognition of scientific societies through the publication of recommendations on the topic.^19^ Research indicates that palliative care integration of palliative care in ESLD is reduced and restricted, usually after exclusion from the liver transplantation list, on the last days of life and in an inpatient setting.^5,20^

ESLD patients are complex, which may explain why outpatient palliative care and home-based palliative care programs are infrequently used in ESLD.^12,21^ A recent study highlighted the possibility of implementing hospital-level care at home for patients with cirrhosis.^22^ However, there is a scarcity of research analyzing the factors that may facilitate home palliative care for ESLD patients, as well as the related outcomes.^23^ Although the majority of ESLD patients continue to die in inpatient care settings,^9^ we believe that home palliative care in ESLD has the potential to reduce costs and increase the likelihood of dying at home. Our primary aim was to determine predictors of home palliative care in ESLD. Secondary aims included examining outcomes of home palliative care for ESLD and identifying factors associated with death at home among ESLD patients.

## Methods

### Study Design

This study was a retrospective longitudinal cohort study of all patients with ESLD followed by a palliative care team between January 1, 2017, and December 31, 2022.

### Patients and Setting

Patients were identified by screening the electronic medical record for the presence of International Classification of Diseases, Ninth Revision, Clinical Modification codes for cirrhosis, chronic liver disease, and indicators of hepatic decompensation. Patients under 18 years old, patients who died from acute hepatic failure or liver transplant, and those with chronic liver disease but who died from a cause unrelated to the evolution of chronic liver disease were excluded. These criteria aimed to minimize selection bias and capture a broad scope of patients.

After excluding patients with ESLD who died from a cause unrelated to the evolution of chronic liver disease and those with missing data (4%, *n* = 3), we categorized total study population into two cohorts: patients who received home palliative care (palliative homecare cohort) and those who did not (no-palliative homecare cohort).

The study setting was an integrated palliative care unit serving a population of approximately 170,000 people. The unit was located within a secondary referral hospital (approximately 380 beds), with full facilities. The unit provided both hospital and community outpatient clinics.

### Data Collection and Outcomes

Data were collected using the hospital's digital information system. We collected information regarding patient demographics: age, sex, and Karnofsky Performance Scale^24^ and liver-specific variables such as liver disease etiology, liver disease complications (ascites, spontaneous bacterial peritonitis, hepatic encephalopathy, gastrointestinal bleeding, hepatorenal syndrome, acute-on-chronic liver failure (ACLF), and hepatocarcinoma), and liver disease severity (Model for End-Stage Liver Disease (MELD)-Na and Child-Pugh scores).^25,26^ The use of these scoring systems was due to their widely use in predicting prognosis in patients with ESLD.

The primary outcomes of interest were predictors of home palliative care in ESLD. Secondary outcomes included results of home palliative care in ESLD (assessing the impact on hospital readmissions through the number of emergency episodes and hospitalizations) and death at home in patients with ESLD.

### Statistical Analyses

Categorical variables were specified as counts and percentages, and continuous variables were specified using mean and standard deviations when normally distributed and as medians and ranges otherwise. Comparisons between categorical variables were performed with the Pearson chi-square test or Fisher's exact test where appropriate, and comparisons between continuous variables were carried out using the Student's *t*-test for normally distributed variables or the Wilcoxon rank-sum test otherwise. Two-sided tests were applied and considered statistically significant when *p*-values were <0.05. Analyses were performed using the Statistical Package for the Social Sciences for Mac (version 26).

### Ethical Considerations

The study protocol conforms to the ethical guidelines of the 1975 Declaration of Helsinki as reflected in a priori approval by the Ethics Committee of Matosinhos Local Health Unit (131/CES/JAS). Being a noninterventional study of deceased patients, written informed consent was waived by the Ethics Committee.

## Results

### General Characterization of the Sample

Between 2017 and 2022, there were a total of 2558 patients followed by our palliative care team, with 78 patients diagnosed with ESLD (3.05%). Two patients with ESLD died from a cause unrelated to the evolution of chronic liver disease, and one patient had missing data. The total study population was 75 patients: 44% (*n* = 33) in the palliative homecare cohort and 56% (*n* = 42) in the inpatient setting (no-palliative homecare cohort). The general characteristics of this population are found in Tables 1 and 2.

**Table 1.** Characteristics of Patients with ESLD Followed by the Palliative Care Team Between 2017 and 2022.

		*n*	%
Gender	Female	17	22.7
	Male	58	77.3
Karnofsky score	0-40	22	29.3
	50-70	43	57.3
	80-100	10	13.3
Etiology of chronic liver disease	Alcohol	57	76.0
	Metabolic dysfunction- associated fatty liver	1	1.3
	Other	1	1.3
	Hepatitis B virus	6	8.0
	Hepatitis C virus	10	13.3
Type of decompensation	Ascites	30	47.6
	Hepatic encephalopathy	15	23.8
	Upper gastrointestinal bleeding	3	4.8
	Spontaneous bacterial peritonitis	3	4.8
	Hepatorenal syndrome	5	7.9
	ACLF	7	11.1
Child-Pugh score	A	8	10.7
	B	38	50.7
	C	29	38.7
Hepatocarcinoma	Yes	57	76.0
	No	18	24.0
Place of death	Ward	50	66.7
	Home	25	33.3
Symptoms of patients with ESLD	Pain	37	49.3
	Asthenia	18	24.0
	Anorexia	12	16.0
	Nausea/vomiting	8	10.7
	Dyspnea	16	21.3
	Restlessness	37	49.3
	Insomnia	2	4.0

**Table 2.** Characterization of age, length of illness, and MELD-Na in the ESLD population and comparison in the home palliative care cohort.

			Home palliative care cohort				
Variable	Total		Yes		No		*t*-Test (*p*)
	$\bar{X}$ ±s	Min-max $\tilde{X}$	$\bar{X}$ ± s	Min-max $\tilde{X}$	$\bar{X}$ ±s	Min-max $\tilde{X}$	
Age	68.00 ± 11.47	38-90 70.00	72.52 ± 10.84	40-90 75.00	64.45 ± 10.79	38-81 67.50	**3.205 (0.002)**
Length of illness (years)	3.79 ± 2.19	1-10 3.00	3.70 ± 2.34	1-10 3.00	3.86 ± 2.10	1-10 3.00	−0.312 (0.756)
Palliative care intervention time (days)	90.45 ± 158.36	1-874 30.00	149.97 ± 196.23	3-874 75.00	43.69 ± 100.60	1-589 10.50	**2.833 (0.007)**
MELD-Na index	18.76 ± 6.01	9-34 18.00	17.45 ± 5.37	10-30 15.00	19.79 ± 6.35	9-34 19.00	−1.687 (0.096)

$\bar{X}$ ± s, mean ± standard deviation; Min-max, minimum-maximum; X ˜, median; *t*-test (*p*), parametric test statistics (significance level) for comparing results between men and women. Statistically significant *p*-values ​​are marked in bold.

The mean age was 68.00 ± 11.47 years, with 50% of patients being up to 70 years old and the majority of patients being male (77.3%; *n* = 58). The majority had a Karnofsky functionality scale of 50 to 70 (57.3%; *n* = 43). Overall, the main cause of ESLD was alcohol (76.0%; *n* = 57), followed by hepatitis C virus infection (13.3%; *n* = 10). Regarding the type of decompensation, it was observed that, overall, the main types were ascites (47.6%; *n* = 30) and hepatic encephalopathy (23.8%; *n* = 15). The MELD-Na was 18.76 ± 6.01, with a median of 18.00 and half had a Child-Pugh class B score (50.7%; *n* = 38). On average, patients had been diagnosed with chronic liver disease for 3.79 ± 2.19 years and a palliative care intervention time of 9.45 ± 158.36 days, with a median of 30 days. The majority of patients (76.0%; *n* = 57) had hepatocarcinoma. The most prevalent symptoms in this population were pain 49.3% (*n* = 37), restlessness 49.3% (*n* = 37), and asthenia 24% (*n* = 18).

### Factors Associated with Home Palliative Care in ESLD

Patients with ESLD in home palliative care had a higher average age of 72.52 ± 10.84 versus 64.45 ± 10.79 years (*T* = 3.205; *p* = 0.002) and a palliative care intervention time of 149.97 ± 196.23 days versus 43.69 ± 100.60 days (*T* = 2.833; *p* = 0.007). Those patients more often presented ascites and hepatic encephalopathy as decompensation (χ^2^ = 11.024; *p* = 0.029) and a diagnosis of hepatocarcinoma (χ^2^ = 7.181; *p* = 0.007; Tables 2 and 3).

**Table 3.** Factors associated with home palliative care in ESLD.

		Home palliative care		Total *n* (%)	χ^2^(*p*)
		Yes *n* (%)	No *n* (%)		
Gender	Female	5 (15.2)	12 (28.6)	17 (22.7)	1.899 (0.266)
	Male	28 (84.8)	30 (71.4)	58 (77.3)	
Karnofsky score	0-40	6 (18.2)	16 (38.1)	22 (29.3)	3.540 (0.176)
	50-70	22 (66.7)	21 (50.0)	43 (57.3)	
	80-100	5 (15.2)	5 (11.9)	10 (13.3)	
Chronic liver disease etiology	Alcohol	26 (78.8)	31 (73.8)	57 (76.0)	0.251 (0.786)
	Other	7 (21.2)	11 (26.2)	18 (24.0)	
Type of decompensation	Ascites	18 (69.2)	12 (32.4)	30 (47.6)	**11.024 (0.029)**
	Hepatic encephalopathy	4 (15.4)	11 (29.7)	15 (23.8)	
	Gastrointestinal bleeding	1 (3.8)	2 (5.4)	3 (4.8)	
	Spontaneous bacterial peritonitis	1 (3.8)	2 (5.4)	3 (4.8)	
	Hepatorenal syndrome	2 (7.7)	3 (8.1)	5 (7.9)	
	ACLF	0 (0.0)	7 (18.9)	7 (11.1)	
Child-Pugh	A	5 (15.2)	3 (7.1)	8 (10.7)	5.418 (0.063)
	B	20 (60.6)	18 (42.9)	38 (50.7)	
	C	8 (24.2)	21 (50.0)	29 (38.7)	
Hepatocarcinoma	Yes	30 (90.9)	27 (64.3)	57 (76.0)	**7.181 (0.007)**
	No	3 (9.1)	15 (35.7)	18 (24.0)	
Patients’ symptoms	Pain	17 (51.5)	20 (47.6)	37 (49.3)	0.112 (0.818)
	Asthenia	12 (36.4)	6 (14.3)	18 (24.0)	**4.938 (0.032)**
	Anorexia	8 (24.2)	4 (9.5)	12 (16.0)	2.979 (0.115)
	Nausea/vomiting	5 (15.2)	3 (7.2)	8 (10.7)	1.244 (0.453)
	Dyspnea	8 (24.2)	8 (19.0)	16 (21.3)	0.297 (0.777)
	Restlessness	9 (27.3)	28 (66.7)	37 (49.3)	**11.474 (0.001)**
	Insomnia	1 (3.0)	2 (4.8)	3 (4.0)	0.144 (0.999)
Local of death	Home	22 (66.7)	3 (7.1)	25 (33.3)	**29.464 (0.000)**
	Ward	11 (33.3)	39 (92.9)	50 (66.7)	

χ^2^(*p*), chi-square independence test statistic (significance level). Statistically significant *p*-values ​​are marked in bold.

Gender (χ^2^ = 1.899; *p* = 0.266), chronic liver disease etiology (χ^2^ = 3.485; *p* = 0.470), and Karnofsky functionality scale (χ^2^ = 3.540; *p* = 0.176) did not show differences between patients receiving homecare and patients in an inpatient setting.

MELD-Na values were identical between patients’ palliative homecare cohort and no-palliative homecare cohort: 17.45 ± 5.37 versus 19.79 ± 6.35 (*T* = −1.687; *p* = 0.096). Also, the severity of the disease according to the Child-Pugh score (χ^2^ = 5.418; *p* = 0.063) and the length of illness 3.70 ± 2.34 versus 3.86 ± 2.10 (*T* = −0.312; *p* = 0.756) were similar.

A lower prevalence of restlessness was documented in patients undergoing home palliative care (χ^2^ = 11.474; *p* = 0.001).

### Outcomes of Home Palliative Care in ESLD Patients

The number of emergency episodes and hospitalizations before and after home monitoring of patients with ESLD were compared. In terms of emergency episodes, there was a marked reduction with home monitoring through palliative care: 2.61 ± 1.71 admissions versus 1.06 ± 1.48 admissions (*T* = 4.507; *p* = 0.000). Regarding the number of hospitalizations, a decrease was also observed: 0.94 ± 0.90 hospitalizations versus 0.58 ± 0.75 hospitalizations, but without significance (*T* = 1.926; *p* = 0.063). It was also found that home monitoring increased the probability of death at home by 66.7% versus 33.3% (χ^2^ = 29.464; *p* = 0.000; Table 4).

**Table 4.** Outcomes of home palliative care in ESLD patients.

Variable	Before home palliative care program	After home palliative care program	*t*-Test (*p*)
	$\bar{X}$ ± s	$\bar{X}$ ± s	
Number of emergencies	2.61 ± 1.71	1.06 ± 1.48	**4.507 (0.000)**
Number of hospitalizations	0.94 ± 0.90	0.58 ± 0.75	1.926 (0.063)

$\bar{X}$ ± s, mean ± standard deviation; *T* (*p*), parametric test statistics (level of significance) for comparing results between patients considered for palliative care and those who were not. Statistically significant *p*-values ​​are marked in bold.

### Death at Home in ESLD

Regarding the place of death of the 75 ESLD patients, 33.3% (*n* = 25) died at home. These patients had a higher mean age 72.20 ± 8.82 years versus 64.40 ± 10.99 years (*T* = 4.267; *p* = 0.000), a longer palliative care intervention time of 178.80 ± 211.78 days versus 46.28 ± 99.67 days (*T* = 2.969; *p* = 0.006), and a lower MELD-Na 16.68 ± 4.74 versus 19.80 ± 6.35 (*T* = −2.171; *p* = 0.033; Tables 5 and 6).

**Table 5.** Factors associated with death at home in ESLD.

		Death at home		Total *n* (%)	χ^2^(*p*)
		Yes *n* (%)	No *n* (%)		
Gender	Female	3 (12.0)	14 (28.0)	17 (22.7)	2.434 (0.151)
	Male	22 (88.0)	36 (72.0)	58 (77.3)	
Karnofsky score	0-40	6 (24.0)	16 (32.0)	22 (29.3)	1.625 (0.450)
	50-70	14 (56.0)	29 (58.0)	43 (57.3)	
	80-100	5 (20.0)	5 (10.0)	10 (13.3)	
Chronic liver disease etiology	Alcohol	19 (76.0)	38 (76.0)	57 (76.0)	0.001 (0.999)
	Other	6 (24.0)	12 (24.0)	18 (24.0)	
Type of decompensation	Ascites	10 (55.6)	20 (44.4)	30 (47.6)	5.940 (0.260)
	Hepatic encephalopathy	6 (33.3)	9 (20.0)	15 (23.8)	
	Gastrointestinal bleeding	0 (0.0)	3 (6.7)	3 (4.8)	
	Spontaneous bacterial peritonitis	0 (0.0)	3 (6.7)	3 (4.8)	
	Hepatorenal syndrome	2 (11.1)	3 (6.7)	5 (7.9)	
	ACLF	0 (0.0)	7 (15.6)	7 (11.1)	
Child-Pugh	A	4 (16.0)	4 (8.0)	8 (10.7)	5.695 (0.058)
	B	16 (64.0)	22 (44.0)	38 (50.7)	
	C	5 (20.0)	24 (48.0)	29 (38.7)	
Hepatocarcinoma	Yes	21 (84.0)	36 (72.0)	57 (76.0)	1.316 (0.390)
	No	4 (16.0)	14 (28.0)	18 (24.0)	
Patients’ symptoms	Pain	10 (40.0)	27 (54.0)	37 (49.3)	1.307 (0.253)
	Asthenia	6 (24.0)	12 (24.0)	18 (24.0)	0.001 (0.999)
	Anorexia	7 (28.0)	5 (10.0)	12 (16.0)	4.018 (0.091)
	Nausea/vomiting	4 (16.0)	4 (8.0)	8 (10.7)	1.119 (0.429)
	Dyspnea	7 (28.0)	9 (18.0)	16 (21.3)	0.993 (0.375)
	Restlessness	10 (40.0)	27 (54.0)	37 (49.3)	1.307 (0.329)
	Insomnia	0 (0.0)	3 (6.0)	3 (4.0)	1.563 (0.546)

χ^2^(*p*), chi-square independence test statistic (significance level).

**Table 6.** Comparison of age, length of illness, and MELD-Na in ESLD patients with death at home.

	Death at home in ESLD				*t*-Test (*p*)
Variable	Yes		No		
	$\bar{X}$ ± s	Min-max $\tilde{X}$	$\bar{X}$ ± s	Min-max $\tilde{X}$	
Age	75.20 ± 8.82	58-90 77.00	64.40 ± 10.99	38-82 67.50	**4.267 (0.000)**
Length of illness (years)	3.60 ± 2.22	1-10 3.00	3.88 ± 2.20	1-10 3.00	−0.518 (0.606)
Palliative care intervention time (days)	178.80 ± 211.78	3-874 87.00	46.28 ± 99.67	1-589 13.00	**2.969 (0.006)**
MELD-Na index	16.68 ± 4.74	10-30 15.00	19.80 ± 6.35	9-34 19.00	−**2.171 (0.033)**

$\bar{X}$ ± s, mean ± standard deviation; *T* (*p*), parametric test statistics (level of significance) for comparing results between patients considered for palliative care and those who were not. Statistically significant *p*-values ​​are marked in bold.

No differences were documented in relation to gender (χ^2^ = 2.434; *p* = 0.151), chronic liver disease etiology (χ^2^ = 4.458; *p* = 0.307), form of decompensation (χ^2^ = 5.940; *p* = 0.260), presence of hepatocarcinoma (χ^2^ = 1.316; *p* = 0.390), and symptoms and Karnofsky functionality scale (χ^2^ = 1.625; *p* = 0.450). Also, the severity of the disease according to the Child-Pugh score (χ^2^ = 5.695; *p* = 0.058) and the length of illness 3.60 ± 2.22 versus 3.88 ± 2.20 (*T* = −0.518; *p* = 0.606) were similar. The prevalence of restlessness (40% vs 54%) and ACLF (0% vs 15.6%) was higher in patients with in-hospital mortality.

## Discussion

This study demonstrated that home palliative care was possible for 44% of the ESLD population, with 1/3 of patients dying at home. There are factors such as advanced age, longer palliative care intervention time, and the presence of hepatocarcinoma that seem to favor home care. We also document that home palliative care in ESLD translates into a greater probability of death at home, as well as a reduction in the number of emergency or hospitalization episodes.

A recent study shows the possibility of implementing hospital-level care at home for patients with cirrhosis.^22^ However, there are very little data on the implementation of home palliative care in patients with ESLD.^27^ With this study we demonstrated that it is possible to implement home palliative care in ESLD and that 44% of the population analyzed had access to this response, especially when in the presence of advanced age, hepatocarcinoma, or ascites. In fact, advanced age has been identified as factors associated with referral to hospice^23,28^ as well as hepatocarcinoma or ascites.^29^ Despite the apparent consistency of data, there are differences between home support through palliative care and hospice care, a reality not always present in all countries. We also document that home palliative care appears to be influenced by follow-up time; patients with a longer palliative care intervention time were more likely to receive homecare. We believe that these data are related to the ability to discuss advance care planning, as has been advocated by some studies,^30^ combined with the fact that these patients possibly have a less severe disease. We must highlight that the MELD-Na score is low in this study population and that ACLF, which has a very poor prognosis,^31^ was more common in inward patients.

We also found in this study that home palliative care translates into a significant reduction in the number of emergency admissions, as well as a trend toward fewer hospitalizations. Although an economic analysis of this impact has not been carried out, we can conclude that reducing pressure at the hospital level will have positive impacts in terms of direct costs. In fact, the data obtained reinforce information from other studies that have demonstrated the positive impact in economic terms of implementing palliative care in ESLD.^14,32^ A previous study also demonstrated that palliative care intervention resulted in increased time to first readmission and more days alive out of hospital.^33^ Another more recent study also showed that the use of palliative care in ESLD resulted in reduction in hospital admissions, highlighting its potential role in reducing unnecessary hospitalizations and in better-coordinated care.^34^

We found that home palliative care increases the probability of death at home. In this study, 33.3% of the population analyzed died at home, a higher value than described by other studies that point to death rates at home of 20% to 25%.^9,23^ In our study, we documented that the presence of advanced age, longer palliative care intervention time, and lower MELD-Na seem to favor death at home. Although some studies suggest that the MELD-Na score^35^ or specific prognostic scales^36^ can induce referral to hospice, the question remains whether this reality can be applied to death at home. Perhaps we can say that the reasons for the probability of death at home are speculative. Possibly, more than clinical data, the cultural framework and the responses of the available health systems are relevant. We cannot assume location of death as a marker of quality of end-of-life care. Some patients may not have access to adequate care, support, or medication access at home. Therefore, certain patients with ESLD may have a quality end of life in an inpatient setting.

We documented that the most prevalent symptoms in this population with ESLD were close to the values described in the review by Peng et al.,^5^ with a considerable percentage of patients experiencing restlessness, possibly as a marker of terminality. We must also notice that the prevalence of restlessness was higher in patients with in-hospital mortality, which likely implies that these patients could not be supported in the home environment.

There are some limitations in this study. First, the sample size is small, with only a few patients receiving home palliative care. This limited number decreases the study's power and may restrict our ability to identify statistically significant differences between the palliative home care cohort and the no-palliative home care cohort. Additionally, the study's single-center and retrospective design introduces potential bias, and the geographic, demographic, and provider practice variations may limit the generalizability of our findings to other centers. Furthermore, as a retrospective study, we were unable to collect data on patient satisfaction or family perspectives, which are crucial elements in advance care planning. Finally, we must acknowledge the high percentage of hepatocarcinoma in this study population (76%).

Despite these limitations, a key strength of this study is that the principles and potential benefits of home palliative care apply across all healthcare settings. This study, one of the few to specifically examine home palliative care for patients with ESLD, confirmed its benefits and has the potential to enhance the integration of home palliative care for patients with ESLD. We must underline the importance of the topic for the scientific community and further enrich the existing knowledge in the area of palliative care and ESLD, as suggested by Patel et al.^18^ We believe that the results of this work will justify further investigations.

## Conclusion

In conclusion, this study analyzed trends of home palliative care in ESLD and outcomes associated with home palliative care. We found a high rate of patients with ESLD followed at home, as well as a death rate at home for this population in line with that described in the literature. Home palliative care and death at home in patients with ESLD differs based on demographics and disease complications, with a positive impact of home follow-up translated into a reduction in hospital admissions. This is one of the few studies on home palliative care in ESLD and aims to underscore the need for further investigation. This study may signal alert and reflection on the potential benefit of home palliative care and improve the care provided to ESLD patients.
